# Clinical correlation of salivary alpha-amylase levels with pain intensity in patients undergoing emergency endodontic treatment

**DOI:** 10.1186/s12903-023-03195-5

**Published:** 2023-08-12

**Authors:** Kavalipurapu Venkata Teja, Sindhu Ramesh, Krishnamachari Janani, Kumar Chandan Srivastava, Deepti Shrivastava, Valentino Natoli, Marco Di Blasio, Marco Cicciù, Giuseppe Minervini

**Affiliations:** 1 Department of Conservative Dentistry and Endodontics, Saveetha Dental College and Hopitals, Saveetha Institute of Medical and Technical Sciences, Chennai, 600077 Tamil Nadu India; 2 Department of Conservative Dentistry and Endodontics, SRM Dental College, Ramapuram, Chennai, Tamil Nadu India; 3 Department of Oral & Maxillofacial Surgery & Diagnostic Sciences, College of Dentistry, Jouf University, Sakaka, 72345 Saudi Arabia; 4 Department of Preventive Dentistry, College of Dentistry, Jouf University, Sakaka, 72345 Saudi Arabia; 5 Department of Periodontics, Saveetha Dental College and Hospitals, Saveetha Institute of Medical and Technical Sciences, Saveetha University, 602105 Chennai, India; 6 Department of Dentistry, School of Biomedical and Health Sciences, European University of Madrid, Madrid, 28670 Spain; 7 Private Dental Practice, Fasano, 72015 Italy; 8 Department of Medicine and Surgery, University Center of Dentistry, University of Parma, Parma, 43126 Italy; 9 Department of Biomedical and Surgical and Biomedical Sciences, Catania University, Catania, 95123 CT Italy; 10 Multidisciplinary Department of Medical-Surgical and Dental Specialties, University of Campania, Luigi Vanvitelli, Naples, 80138 Italy

**Keywords:** Endodontics, Pain, Pain management, Root canal therapy, Salivary alpha-amylases

## Abstract

**Background:**

Pain is usually subjective and thus it is challenging to describe its characteristics such as nature, intensity, and origin. Non-invasive methods such as assessing salivary alpha-amylase (SAA) may aid the practitioner to evaluate the pain intensity. Hence, the current study aimed to correlate the levels of SAA with the pain intensity in patients presenting with varied endodontic pain levels.

**Methods:**

Sixty patients who presented with varied intensities of endodontic pain were selected for the present study out of which seven patients were excluded/dropped, leaving a total sample of fifty-five patients for assessment. Mandibular molar with symptomatic irreversible pulpitis without periapical pathology were included in the study. A 5ml of un-stimulated was obtained from the patients, following which the local anesthesia was administered. Root canal treatment was then performed and the pain scores at pre-operative and post-operative were recorded. Additionally, salivary samples were collected after emergency endodontic treatment and sent for sialochemical analysis. IBM.SPSS statistics software 23.0 was employed to assess the obtained data.

**Results:**

A statistically significant drop in the pain score (P < 0.001) and SAA levels (P < 0.001) were observed post-operatively in the contract to pre-operative state. A strong positive correlation was reported between SAA levels and pain scores in patients undergoing emergency endodontic treatment at both time intervals namely pre-operative (P < 0.001) and post-operative (P < 0.001).

**Conclusion:**

The results of this preliminary showed a strong association between the pain score and SAA levels in patients undergoing an emergency endodontic treatment.

## Introduction

Pain is an unpleasant sensation which is most often related to potential injuries, due to psychosocial issues, or direct from other areas [1]. Most often it isn’t easy to assess its original cause and diagnose the clinical condition [2]. Pulpal damage is usually followed by dental pulp exposure by caries or acute trauma, inducing intermittent or spontaneous pain. Significant changes in the temperature cause longstanding and severe pulpal pain that prolongs the removal of stimuli. The severity of the pulpal pain is mainly due to the inflammation of the surrounding periodontal ligament and bone [3]. Frequently patients seek endodontic treatment due to tooth pain. Tooth pain is unbearable for patients leading them to visit a dentist or a specialist. Endodontic procedures have an important role in restoring and treating a tooth which is affected irreversibly by bacterial invasion and infection. Endodontic treatments majorly aim at thorough cleaning and removal of the infected tissue and pulpal contents from the prepared root canal and sealing the space to prevent microbial ingress both from coronal and periapical areas. Pain resolution is absolute and immediate after an emergency endodontic treatment. But, the pain relief may not be instant and absolute most of the time [4, 5]. Although in 30% of the situations, the pain relief is better, patients still experience moderate to severe post-endodontic pain after root canal therapy. The main reason for such experienced pain is due to the inherent inflammatory effects of root canal infection [6]. Root canal therapy should majorly consider the pain reduction to the patient and long-term complications which can occur after the treatment. Most of the time patients experience pain immediately after the endodontic treatment after the anesthesia wears off. The experienced pain is usually severe at 12 and 24 h, which reduces slowly within 3 to 7 days [6].

The pulpal and periapical pain is majorly stimulated by various physical and physiological factors, leading to nerve sensitization leading to a painful response [7]. Various endogenous inflammatory mediators stimulate the nociceptors, which induce pain [8]. The nociceptors usually follow tissue damage or injury, which seems to be an inherent response to an innate immune reaction. The pain threshold of the patient is reduced instantly when the prostaglandin conversion happens. Arachidonic acid converts into cyclooxygenase enzymes type 1 and 2 causing an afferent nociception and decreased pain threshold [9]. Hence the pharmacological strategies using the pain killers target limiting the prostaglandin production and conversion thereby reducing the sensitization of the nerve fibers [10].

Pain is a complicated mechanism, which is difficult to understand. It’s highly subjective and varies from patient to patient. Multiple patient-based, and operator-based factors play a vital role in perceiving pain. The real pain can never be quantified. The patient’s threshold to perceive real-time pain varies based on both physiological and various psychological factors too [11]. The day-to-day dental procedures induce a painful response in the patients. There are other various conditions, which impact and induce anxiety and stress in the patients[12, 13] and the reported tendency is higher in patients with temporomandibular disorders [14–18]. The reported incidence of this pulpal and periapical pain is severe following a root canal treatment [19]. Various factors have a role in pain induction after endodontic therapy. Although the periapical extrusion of the debris, canal contents and irrigants is one reason for such a painful response, various other operator-based factors also induce such occurrence [20]. Different strategies such as pharmacological and non-pharmacological modalities play a key role in pain reduction. Non-steroidal anti-inflammatory drugs are mostly used to treat endodontic-related pains. They act by reducing the prostaglandin levels and synthesis and finally reducing the cyclooxygenase enzyme levels [21]. The patient-based studies showed that non-steroidal anti-inflammatory drugs are widely used for treating root canal-related pain. However, the results are controversial. Many showed a beneficial response from the patients, but few oppose their usage [22, 23]. Recent literature showed a wide interest in various other drugs for treating endodontic pain. Drug combinations have also been tried to enhance pharmacological actions, thereby reducing the pain response [24].

Pain is usually subjective, and it is challenging to describe its nature, intensity and identify the origin of pain. The use of non-invasive techniques, such as measuring salivary alpha-amylase, may assist the physician in determining pain severity [25]. Saliva constitutes many proteins, and alpha-amylase contributes to 50 to 60% of total salivary proteins [26].

Amylase is an important enzyme and activates by the sympathetic reactions to psychosocial stress [27, 28]. The plasma catecholamine levels are correlated with the salivary amylase levels. But it cannot be regarded as a genuine, precise indicator [27, 28].

Pain activates the sympathoadrenal medullary (SAM) activity and the hypothalamic-pituitary-adrenal axis. Exaggerated levels of the salivary alpha-amylase are seen in psychosocial stress, which indirectly reflects the SAM activity. As painful stimuli activate the SAM system and hypothalamic-pituitary-adrenal axis, pain-associated stress is related to salivary alpha-amylase levels [2, 29, 30]. Epinephrine and norepinephrine are released from the adrenal medulla whenever the autonomic nervous system gets activated [31].

With norepinephrine, the parotid and submandibular glands’ acinar cells secrete more SAA [32]. The level of SAA reflects the autonomic nervous system (ANS) activity, and measuring its salivary level is an easy and non-invasive measure of ANS activity compared to measuring actual catecholamine levels in serum [32]. Various stresses like exercise, cold exposure, and hypertension increase SAA levels. The SAA levels also seemed to fluctuate during the day in a classic pattern and have a standard circadian rhythm [32].

Salivary amylase levels were significantly associated with experiencing chronic pain, according to Shirasaki et al., 2004 [33]. Pain scales are helpful in the assessment of pain and treatment response [34]. The intensity and other aspects of pain are generally measured by pain scales, which are divided into three categories: self-report, observational and physiological data. The most sensitive subjective scale for assessing pain severity is the visual analogue scale (VAS) [35].

Hence, the present study aimed to determine the association between salivary alpha-amylase and the intensity of pain. The previous investigation examined the relationship between salivary alpha-amylase levels and pain in patients who reported symptomatic irreversible pulpitis [36]. This research had a unique approach in evaluating the patients presented for seeking endodontic treatment with varied reasons and varied intensities and severities of pain. Usually, it is reliable to assess and generalize its activity in all clinical endodontic conditions where patients present with higher pain intensity, rather than restricting its assessment to specific clinical situations. The null hypothesis was patients presenting with endodontic pain of varied intensities did not significantly differ in SAA levels or VAS scores.

## Materials and methods

### Study and sample characteristics

**Fig. 1:**
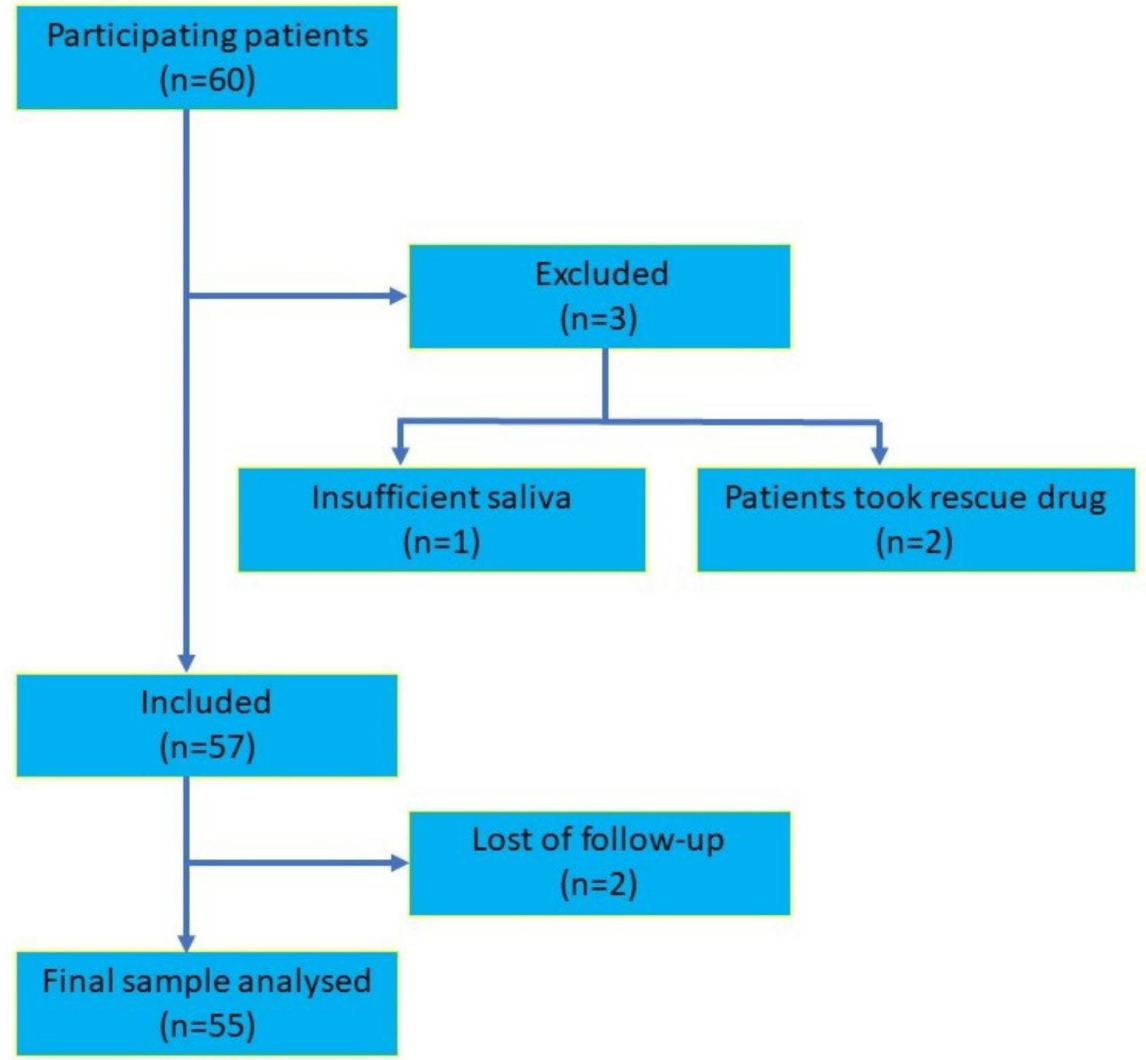
Patient recruitment Flowchart

The institutional ethical committee had approved the protocol for the present prospective study (IHEC/SDC/FACULTY/21/ENDO/135, dated 16/6/2021). The current study has been registered on clinical trials.gov with the registration number REF/2023/06/068283. A total of 60 systemically healthy ASA I (​as per the physical status classification system of the American Society of Anesthesiologists) individuals aged 18–55 years who presented varied pain intensities were selected for the study. Three patients were excluded before obtaining the salivary samples, as two of them took a rescue drug, and one had insufficient saliva. An additional two patients were lost during follow-up visits, with a remaining sample of 55 patients considered for the study (Fig. 1). Examiner 1 (K.V.T.) explained the entire treatment procedure including the study objectives to the patient before getting their informed consent. After obtaining the VAS scoring, the patients were then assigned to endodontic therapy.

### Inclusion and exclusion criteria

The inclusion criteria for the study were as follows: Systemically healthy ASA I male and female individuals aged 18–55 years. Patients diagnosed with symptomatic irreversible pulpitis involving mandibular first molars without any associated periapical pathologies were selected. The other criteria included patients who were not under any medication, and patients presenting with varied intensities of endodontic pain. Pregnant and nursing women, women in their menstrual cycles, patients on inhaled steroids or any other analgesic for endodontic pain, antidepressants, antihypertensives, or antipsychotic drugs, xerostomia, smokers, drinkers, periodontitis, oral lesions, and users of any illicit drugs were all excluded.

### Sample size determination

The sample size was determined using the G Power 3.1 version. Considering the objectives of the study, the sample size was computed for the difference between two paired means (pre and post-operative), with an effect size of 0.5, α of 0.05 and CI of 95%. The total sample estimated was 54. The overall sample was adjusted to 60 to compensate for the loss of follow-up and other unexpected situations.

### Clinical protocol

Patients with mandibular molar presenting with symptomatic irreversible pulpitis were only selected. The tooth in question satisfied the criteria for the diagnosis of symptomatic irreversible pulpitis. The clinical diagnosis was determined based on prolonged symptomatic response to cold and electric stimuli with no tenderness on vertical or lateral percussion. The radiographic diagnosis of an intact lamina dura with no evident periapical widening associated with root apex was confirmative. After the preoperative diagnosis and selection of included subjects for participation in the present study, informed consent is obtained from the patients before allocation. Once the diagnosis was confirmed, each included patient was assessed for an initial baseline pain score using a visual analogue scale. Examiner 1 (K.V.T.) who obtained informed consent from the patient performed the clinical examination and wrote the diagnosis of the endodontic condition. In the interview section, the examiner recorded the details about the history of the pain including the patient’s previous treatments. He also charted the characteristics of pain including onset, type, nature, site, source, and aggravating and reliving factors. Before the anesthetic administration, a visual analogue scale (VAS) was used to measure the intensity of the pain and scores were recorded. A 5ml of unstimulated saliva was obtained from the patient in a plastic container with graded markings on it. The patient was instructed to sit upright with the neck bent forward, and the unstimulated saliva was collected using the spitting method [37, 38].

Once the diagnosis was confirmed and VAS scoring was obtained, the patients were then randomly allocated into two groups by a head nurse using the opaque sealed envelope method. The patient and the operator were blinded by the pre-operative VAS scores.

Once examiner 1 (K.V.T.) collected the pre-operative sample, the local anesthesia was administered by a blinded operator (S.R.). The entire treatment procedure was performed by a single operator (S.R.) blinded to the pre-operative data. Once the subject allocation was done, each subject was anesthetised by the standardised inferior alveolar nerve block, using 1.8ml of 2% lidocaine with 1:200.000 epinephrine and an additional second dose is administered if no profound anesthesia was obtained. Before injection, sterile gauze was used to dry the site of injection. Topical anestheic 20% benzocaine was applied using a sterile cotton applicator tip. Once the negative aspiration was performed, the solution was deposited at a rate of 1ml/min using a 27-gauge long needle. After 15 min waiting period, the patient was assessed for profound lip numbness. Once the lip numbness was confirmed, the rubber dam isolation was done and treatment was initiated. Patients, who experienced a failure of anesthesia, were administered supplemental infiltrations and intraligamentary injections. Intrapulpal anesthesia was a final resort in patients who experienced intolerable pain during pulp extirpation or instrumentation.

As mentioned, after the confirmation of profound anesthesia, the rubber dam isolation was carried out and the standardized access cavity was prepared under an operating microscope (CARL ZEISS). Once the apical patency was maintained, the working was assessed using an apex locator. The working length radiograph was taken as a confirmative. Once the working length was established, the treatment protocol was initiated. Standardized instrumentation was carried out by hybrid technique using hand K-files and Protaper gold rotary files (Dentsply Tulsa, USA). Apical preparation was established to at least three sizes greater than the initial apical binding file. In due course of instrumentation, intermittent irrigation was carried out using 5% sodium hypochlorite (NaOCl) (Parcan, India) with a 30-gauge side vented needle (NaviTip, Ultradent Products, South Jordan, UT, USA). 10ml of 5%NaOCl was used for each canal. The needle was oscillated at a frequency of 1 Hz, at an amplitude of 3 mm continuously until the end of the irrigant delivery. After the complete instrumentation, irrigation was carried out using 4ml of 5% NaOCL and 5ml of 17% ethylene diamine tetra acetic acid (EDTA), (MD Cleanser, MetaBiomed, India). The final rinse was carried out using 5 ml of distilled water. The entire syringe needle irrigation was carried out using a 30-gauge side vented needle attached to a 5ml syringe barrel. At the end of instrumentation and manual irrigation, activation was carried out using passive ultrasonic irrigant activation (PUI). An amount of 1 mL of 5.25% NaOCl was activated for 20 s, which was performed for 3 consecutive cycles using an IRRI S ultrasonic tip (VDW), attached to an ultrasonic device (Ultra Device, VDW), at a power setting at 30, placed 1 mm short of the working length. Following the activation, the final rinse was carried out using 4 mL of 5.25% NaOCl, 5 mL of 17% EDTA, and 5 ml of distilled water.

Canals were dried, the sterile cotton pellet was placed, and the closed dressing was given using resin-modified glass ionomer cement RMGIC (GC Fuji CEM 2, GC America, Alsip, IL, USA). No intracanal medicament was placed, and the obturation was carried out on the next visit. Patients were requested to wait at the reception and were asked to report back to the operatory after 3 h. In the meantime, they were monitored in the outpatient department and not asked to take anything per oral. A resident doctor (who is not a part of the study and not aware of the pre-operative VAS scoring) was instructed to prescribe 600 mg of paracetamol as a rescue drug and patients were told to report if they had intolerable pain. Patients with unbearable pain and who have consumed the rescue drugs were excluded from the present study.

After 3 h post-operatively, the VAS scoring was again recorded by examiner 2 (K.J.). Later, the salivary samples were collected, and the patients were dispersed from the clinic. By having different examiners (K.V.T and K.J.) for recording VAS scores at different intervals namely pre-operative and post-operative, the methodology bias was eliminated. Additionally, the operator (S.R.) was also not informed about the VAS scores. Lastly, while recording the post-operative VAS score, the patients were not reminded about their pre-operative score. Hence, all efforts were made to maintain double blinding.

The present study was conducted for 60 days in the Department of Conservative and Endodontics. The experimental protocol was such that only one patient was evaluated in a day to prevent the disparity in results. The treatments started on or before 10 AM. Patients were not included in the study if they reported in the morning after 10:30 AM. The research was scheduled for the next day if the patient reported late. The reason was to ensure that a sufficient postoperative time limit was available as none of the patients were allowed to take anything per oral during the postoperative monitoring. The evidence from the literature shows that there is no role of the circadian rhythm, daily variation in a week, or gender difference in SAA levels under resting conditions [39]. Hence, there was a minor role of circadian rhythms in selecting the patients for the present study.

### Sialochemical analysis

**Fig. 2:**
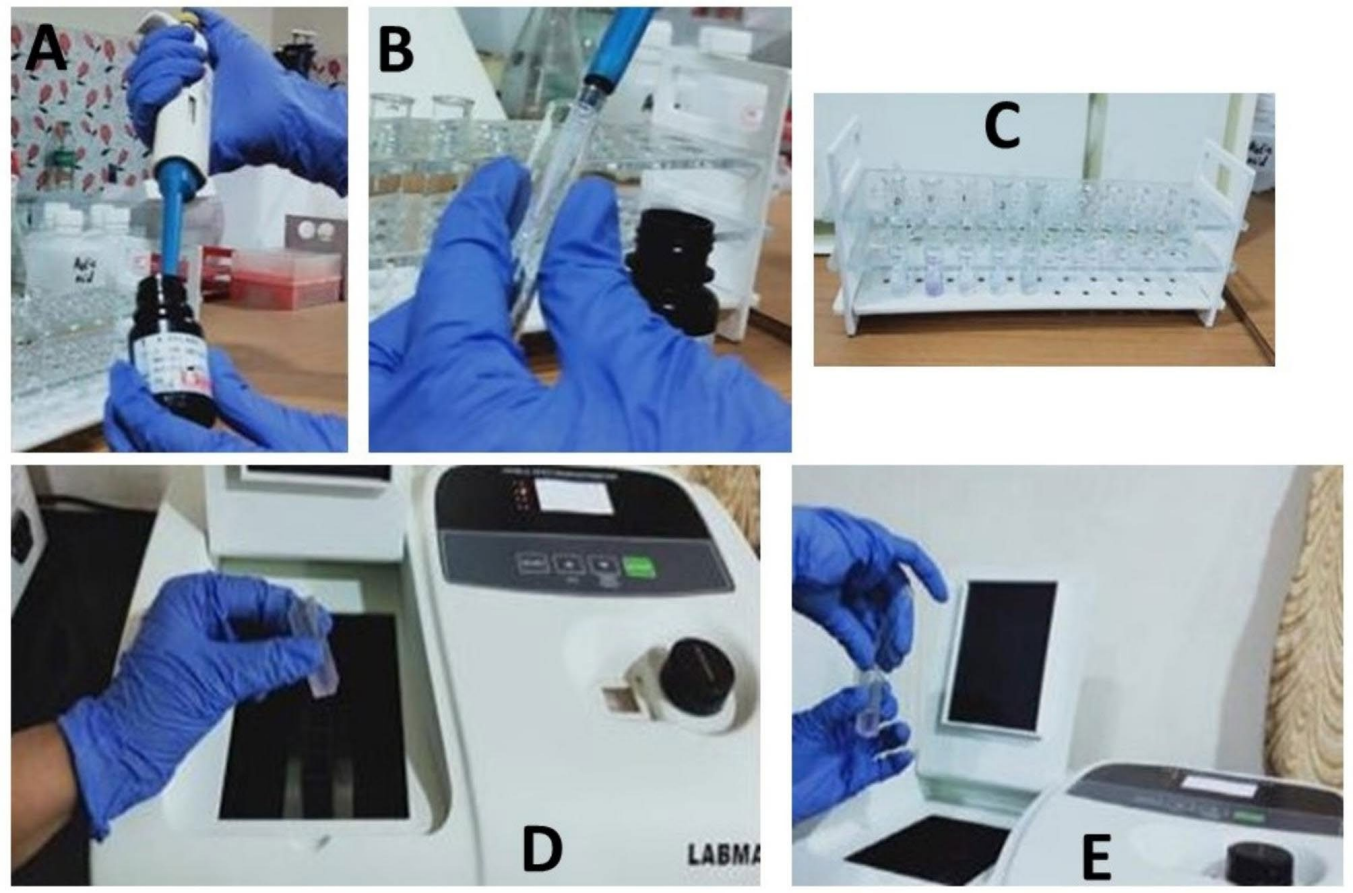
(A-E) -Illustrating the entire protocol of assessment of Salivary Alpha-Amylase. (A) – Collection of reagents; (B) – Addition of reagent to salivary sample; (C) Color change of salivary sample is evident after the addition of reagent; (D & E) – Assessment of color change in the spectrophotometer

The samples were immediately transferred for sialochemical analysis. To obtain pure saliva, the samples were centrifuged for 3–5 min using (KD2-TDSA by Nantong Hailum Bio-medical Apparatus Manufacturing C., Ltd. in Haimen, Jiangsu, China). The SAA level was assessed using a biochemical kit (Liquizyme, Alpha-amylase kit, direct substrate method, BEACON, India) and a spectrometer (LABMAN, India) at a wavelength of 590 nm (Fig. 2 – A-E). A coloured solution of chloro-p-nitrophenol is formed when alpha-amylase reacts with the chromogenic material. The level of enzymatic activity is proportional to the darkness of the substance produced.

### Statistical analysis

With the aid of IBM.SPSS statistics software 23.0, the obtained data were examined. For categorical variables, frequency analysis, percentage analysis, and mean and standard deviation were used to describe the data using descriptive statistics. The paired sample t-test was employed to determine the significant difference between the bivariate samples in paired groups. Additionally, Pearson correlation analysis was carried out to evaluate the association between the variables. The probability value of 0.05 was regarded as a significant level.

## Results

**Fig. 3:**
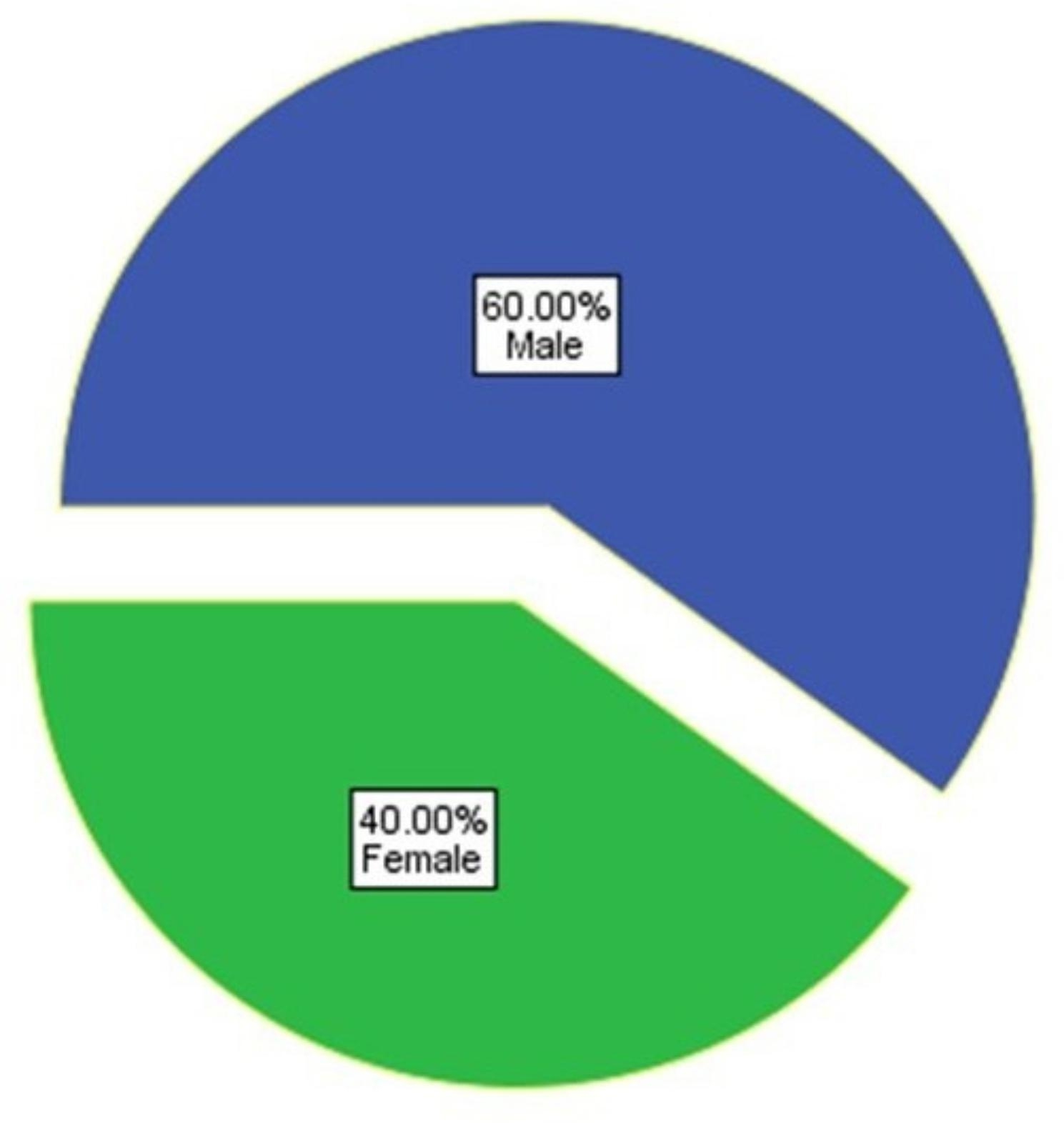
Gender distribution of the sample

**Fig. 4:**
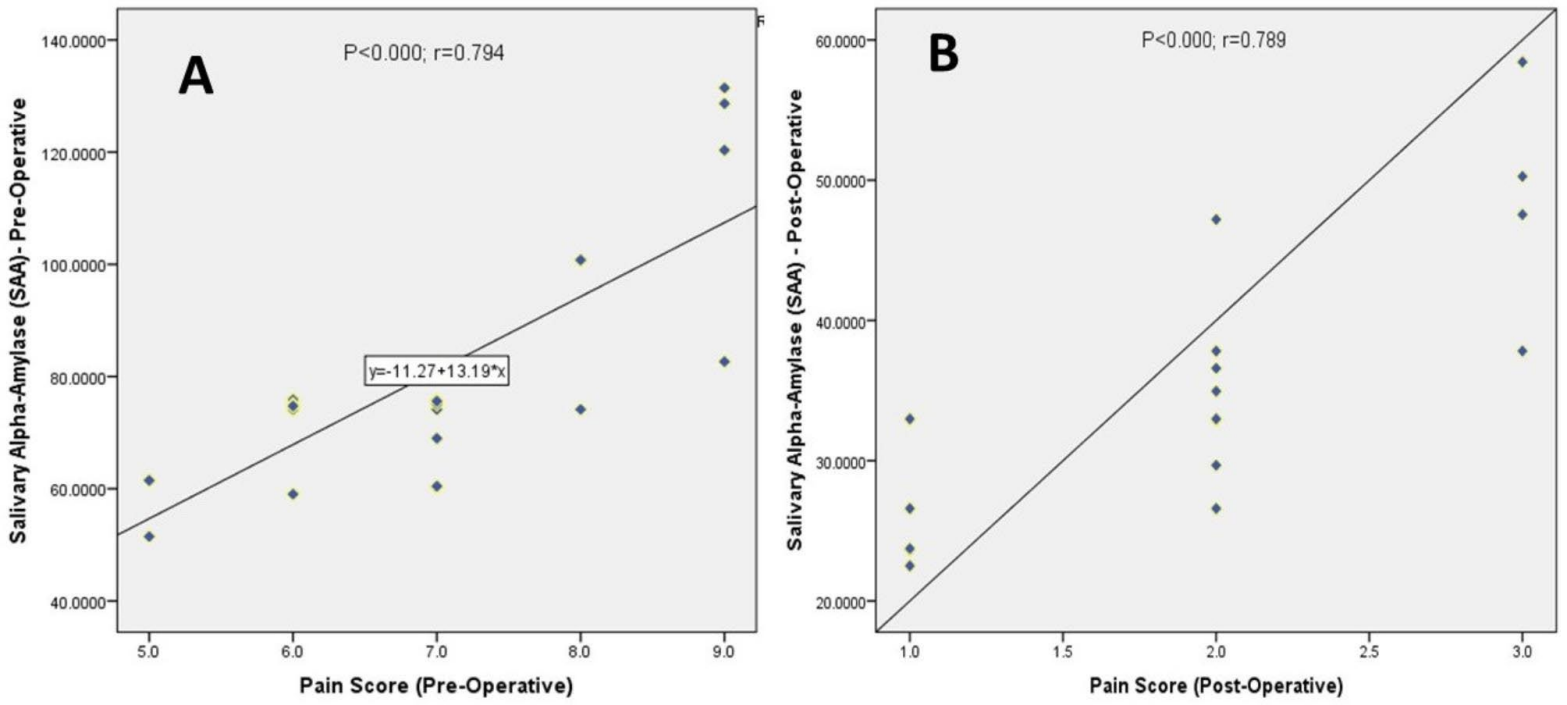
(A & B): Scatter plot depicting association between pain scores and SAA pre-operative (A) and post-operatively (B)

Gender-wise comparison for the patients selected for the study is depicted in pie chart 1 (Fig. 3). A statistically significant drop in the pain scores (*P* < 0.001) and reduction in the salivary alpha-amylase levels (*P* < 0.001) was observed post-operatively when compared with pre-operative status (Table 1). Correlation analysis was performed to assess the association between the variables. A positive correlation was reported between the two parameters (*P* < 0.001) in pre- and post-operative state, which was statistically significant (Fig. 4).

**Table 1:** Comparative analysis of pain intensity and salivary alpha-amalyase levels at different time intervals

Parameter	Interval	Mean ± SD	P value
Pain	Pre-operative	6.83 ± 1.17	0.000*
	Post-operative	1.73 ± 0.69	
SAA	Pre-operative	78.85 ± 19.55	0.000*
	Post-operative	33.07 ± 8.68	

Note: SAA- salivary alpha-amylase; *p < 0.001

## Discussion

The ultimate goal of endodontic therapy is to completely disinfect, clean and shape the canals to receive the obturation that gives a three-dimensional seal from the oral and the periapical fluids. In due course of achieving these goals, various untoward consequences are encountered, which might or might not be in control of the operator. Taking all these into consideration, from a clinical standpoint an endodontic procedure should induce minimal or no pain from starting of the procedure till the end. So, a clinician should not only concentrate on achieving the maximal debridement efficiency and bacterial removal, but also emphasis should be made on achieving the pain control, during the entire duration of the procedure and also postoperatively.

Pain management is considered a key factor, for successful endodontic therapy. Endodontic literature has not only concentrated on the procedure as such but pain management was also considered as a prime and foremost factor for therapeutic success [5]. When pain management is considered, studies concentrated basically on intraoperative pain management[40–44] and postoperative pain management [4, 45, 46]. Studies assessing intraoperative pain management, basically concentrated on assessing the anesthetic efficacy and administering various drugs preoperatively [47–53]. Whereas, studies assessing postoperative pain mostly concentrated on administering drugs preoperatively or postoperatively [10, 54–57].

Postoperative pain control is also considered as important as intraoperative pain management. Especially when pain is considered, it’s multifactorial. One cannot imply a single reason for the causation of pain. Especially this regard is more important with single-visit root canal therapies as compared to multi-visit. Literature shows that single-visit root canal treatments are associated with more post-operative pain as compared to multiple visits [58, 59]. When the literature on pain control is evaluated, studies were constricted on the usage of drugs in postoperative pain management [10, 54–57]. Most of the studies concentrated on pain management using NSAIDs after pulpectomy or root canal preparation, where drugs were prescribed preoperatively[60–64] or postoperatively [65–72]. But, studies mainly concentrating on the usage of drugs in pain management in single-visit root canal treatment are scarce.

[23, 55, 73–75] Very few studies have evaluated especially on NSAIDS on postoperative pain management, especially in single visits [23, 73, 74]. The literature is scarce on the administration of non-steroidal anti-inflammatory drugs before or after the treatment. Even the literature is not clear on the role of biomarkers in assessing the pre and post-endodontic pain evaluations, especially in regards to the single visit root canal treatments.

It is clear from the current study that there was a positive association of innate salivary biomarkers (alpha-amylase) with the pain scores compared. Previous endodontic literature on evaluating these innate biological markers is scarce. According to our knowledge, there was only one study by Ahmadi et al.,[36] that prospectively examined the association between the presence of symptomatic irreversible pulpitis and salivary alpha-amylase levels.

Endodontists are primary care providers before interventional specialists. So, as a primary care provider, the major responsibility is to provide pain-free treatment, especially in patients undergoing emergency endodontic therapy. Hence, it’s appropriate for a primary care provider to assess and evaluate the new avenues of biomarkers and their clinical applicability in therapeutic endodontics.

None of the studies to date, in endodontic literature, have evaluated the present-mentioned protocol. Especially when consideration has to be given, there is scarce knowledge on the evaluation of patients’ pre and postoperative pain levels. In a clinical scenario, we usually tend to prescribe analgesics routinely and collect the postoperative pain scores after the treatment procedures. This is the routine protocol being followed for decades, to quantitatively assess the patient’s pain scores. Which may not be a replicate of a true condition and it’s more subjective than an objective correlation.

Hence, the present study laid a unique perspective in the endodontic field, to assess the pain by clinically correlating the subjective pain scores with an innate biomarker evaluated. When the results of the present study are critically appraised, there was no gender-related bias or preoperative data-related bias. The reason for selecting the patients with mandibular molars, especially with baseline pain scores was because the reported postoperative pain score is more in patients who present with preoperative pain. This could be because of the activation of nociceptors, which leads to central sensitization [23]. Hence, it would be appropriate to select these patients.

When the specific biomarker evaluation is compared, many studies in pediatric literature have evaluated the correlation of the SAA levels with innate correlates such as heart rate and blood pressure in children undergoing dental treatment, to evaluate the anxiety levels [76–78]. The interest in evaluating the SAA levels in patients undergoing extraction also increased in recent days and a positive correlation was also found in comparing the anxiety levels during and after the extraction [78].

When consideration has to be given to other specific biomarkers in dentistry, literature is sparse on especially substance P, which is a true positive correlate to pulpal pain. To our knowledge, only one clinical study by Ahmad et al., found a positive correlation and increased salivary substance P concentrations in patients presented with dental pain [79]. But the literature is vast on salivary cortisol as a biological evaluator, especially for dental stress levels [76–78, 80–82]. Although the assessment of cortisol and substance P is more appropriate, the cost factor should also be taken into consideration. Hence, economically, assessment of SAA levels would be beneficial both in collection and correlation.

When the standardization of the study is analyzed, the teeth selection and the specific preoperative condition were similar for all the patients included in the study. To exclude other possible reasons which might interfere with the inference of the study measures were taken to select the patients with higher baseline pain scores, patients were selected such that they did not consume any prior analgesic before the intervention, patients with additional teeth presenting with pulpal and periapical pathosis, patients under any medication or analgesic intake for pain management or any other medical condition, patients with multiple teeth requiring endodontic treatment were excluded. Only patients categorized under ASA I was only selected for the present study.

The reason for mandibular molars and especially with higher baseline pain scores for the present study was mainly because; the literature shows that, the reported postoperative pain was severe in these teeth [83]. When selecting the patients, it is appropriate to choose the patients, with higher baseline pain scores, as there is an increased activation of nociceptive impulse, leading to higher chances of postoperative pain in such cases [4, 83]. The treatment protocol was standardized for the present study and was similar in both groups.

When the limitations of the present study are considered, the protocol might not be feasible in a true clinical scenario. The patients selected in the present study were only ASA I, patients. However, usually, the patients attending the dental clinic are systemically compromised most of the time. When consideration has to be given to preoperative analgesic intake, most of the patients consume one or the other analgesic before intervention. The sample size of the present study is insufficient to give generalized statements. Hence, future trials have to concentrate on increased sample size along with using additional biomarkers for evaluation.

## Conclusions

Current study revealed a positive association between SAA levels and pain scores in patients undergoing emergency endodontic treatment. Hence future studies should concentrate more on this biological marker to assess its role in endodontic pain.

## Data Availability

The data will be available on reasonable request from the corresponding author.

## Declarations

### Clinical trial registry No

REF/2023/06/068283.
